# Measles Outbreak Worsens in Pakistan

**DOI:** 10.12669/pjms.42.6.19511

**Published:** 2026-06

**Authors:** Muhammad Zain Sultan Meo, Muhammad Omair Sultan Meo

**Affiliations:** 1 Muhammad Zain Sultan Meo, College of Medicine, Alfaisal University, Riyadh, Saudi Arabia; 2 Muhammad Omair Sultan Meo, College of Medicine, Alfaisal University, Riyadh, Saudi Arabia

Measles is a highly contagious, serious airborne disease caused by a virus that can lead to severe complications and death.^1^ After the exposure, between 10 and 14 days, disease begins with a prodromal phase that includes fever and the “3 Cs” clinical symptoms: cough, coryza, and conjunctivitis. This phase lasts for 2-4 days. Koplik spots, small white spots on the buccal mucosa, are not always present, but may appear 1-2 days before the onset of rashes and disappear for an additional 1-2 days. The measles rash begins on the face and spreads to the head, trunk, arms, and legs. The patient transmits the virus four days before and four days after the eruption of the rashes.^2^

**Fig.1:**
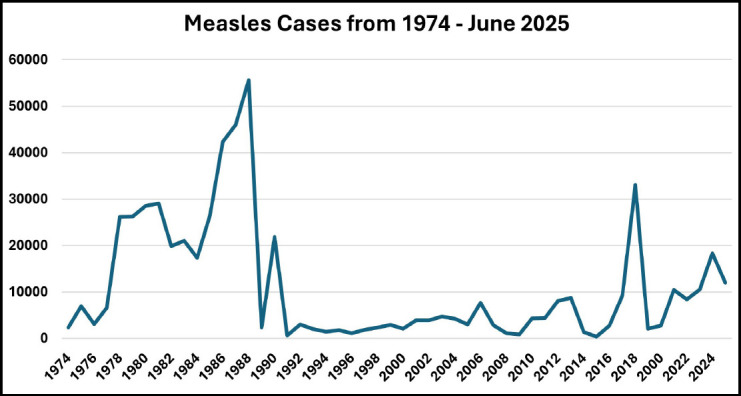
Measles cases in Pakistan from 1974 to June 2025.

As per data from, Our World in Data^3^ and the World Health Organization^4^ in Pakistan the total number of measles cases reported from 1970 to June 2025 is 569364 (Fig.1). Although, Pakistan launched largest campaign of immunization to eradicate measles and immunized over 90 million children against measles and rubella, still, the incidence of the disease is not declining.^5^

This year, April 17, 2026, the Centres for Disease Control and Prevention (CDC) reported that measles can cross borders and cause outbreaks from one country to another among the people who are unvaccinated or under-vaccinated. CDC also highlighted that the top 10 countries with measles outbreaks are India 15,750; Yemen 11,085; Pakistan 8,721; Mexico 8,005; Angola 7,373; Indonesia 5,822; Kazakhstan 5,599; Cameroon 5,109; Sudan 3,689; and Lao People’s Democratic Republic 3,304.^6^

In Pakistan, about 4,541 measles cases were found in the first quarter of the year 2026, with highest number of cases in Khyber Pakhtunkhwa 1,712; followed by Punjab 1,198; Sindh 1,183; Baluchistan 197; Azad Jammu and Kashmir 151; Islamabad 55; and Gilgit-Baltistan 45. Moreover, about 71 children with measles died across the country during the first four months of year 2026, Sindh accounting highest deaths 40: followed by 12 deaths each in Punjab and Khyber Pakhtunkhwa, and 04 in Baluchistan.^7^

Worldwide, measles vaccination prevented approximately 59 million deaths between 2000 and 2024. In 2024, an estimated 95000 deaths occurred globally, mostly among unvaccinated or under-vaccinated children under the age of 5 years. The majority of deaths were due to complications, including pneumonia, encephalitis, and brain damage. The measles morbidity and mortality associated with vaccine hesitancy.^1^

Enhanced research during measles outbreaks is crucial to address gaps in population immunity and improve vaccine coverage. Moreover, timely diagnostic tests could be used routinely as part of measles regional surveillance, improving the timing of measles outbreak responses. In Pakistan, efforts have been made to improve vaccine delivery. There is a further need to educate the people about the significance of vaccines to reduce hesitancy and the burden of measles in Pakistan.
